# Hemodynamic effects of cycle ergometry and low-intensity handgrip in patients with pulmonary hypertension

**DOI:** 10.3389/fmed.2026.1727173

**Published:** 2026-02-25

**Authors:** Alexander Rueß, Stefan Stadler, Tobias Stark, Eugen Berezucki, Tobias J. Lange

**Affiliations:** 1 Department of Internal Medicine II, University Medical Center Regensburg, Regensburg, Germany; 2 Department of Internal Medicine II, District Hospital Bad Reichenhall, Bad Reichenhall, Germany

**Keywords:** diastolic function, exercise, isometric handgrip, pulmonary circulation, right heart catheter

## Abstract

**Introduction:**

In pulmonary hypertension (PH), the hemodynamic response to exercise during right heart catheterization (RHC) can unmask latent left heart disease, is associated with prognosis, and can facilitate the understanding of patient’s symptoms and the pathophysiology of heart failure. As cycle ergometry isn’t widely available, we compared the hemodynamic effects of ergometry with isometric handgrip exercise.

**Methods:**

This prospective study included 50 consecutive patients (mean age 68 ± 12 years, 54% female) with suspected or confirmed PH who underwent RHC. In a semi-recumbent position, hemodynamic parameters were recorded at rest and during both randomized exercise modalities, each sustained for a mean of 6–7 min and separated by another resting phase. Cardiac output was determined by thermodilution. Handgrip pressure was set to 20% of maximal force, while cycle ergometry was performed at a constant work rate between 10 and 50 watts.

**Results:**

Both modalities induced significant changes of hemodynamic parameters compared to baseline, which were considerably smaller during handgrip. Mean pulmonary arterial pressure increased by 14 mmHg under cycling vs. 4 mmHg under handgrip (*p* < 0.001), pulmonary artery wedge pressure (PAWP) by 5 mmHg vs. 2 mmHg (*p* < 0.001), and cardiac output by 1.5 l/min vs. 0.2 l/min (*p* < 0.001). In patients with PAWP ≤ 15 mmHg at rest, an increase > 25 mmHg occurred in two cases during cycle ergometry but in none during handgrip exercise.

**Discussion:**

Compared with cycle ergometry, low-intensity isometric handgrip exercise at 20% of maximal force produces significantly smaller alterations in hemodynamic parameters measured by RHC in PH patients, and thus is not suitable as an adequate substitute for the established exercise modality.

## 1 Introduction

Pulmonary hypertension (PH) is a serious condition characterized by elevated mean pulmonary artery pressure (mPAP) > 20 mmHg at rest, that can lead to right heart failure and death (1). The diagnosis of PH requires invasive right heart catheterization (RHC), which allows direct measurement of pressures in the right atrium, the right ventricle, and the pulmonary artery, as well as pulmonary arterial wedge pressure (PAWP), which reflects left atrial pressure. The PAWP at rest is used to differentiate between precapillary (PAWP ≤ 15 mmHg) and post-capillary PH (> 15 mmHg), which has implications for treatment. In addition, for calculation of pulmonary vascular resistance, the determination of cardiac output (CO) is mandatory. While the gold standard for CO measurement is the direct Fick method, this is unfeasible for clinical practice as it requires a direct measurement of oxygen consumption using a Douglas bag. Therefore, and because the indirect Fick method of CO determination is more inaccurate, the use of the thermodilution method is recommended (1). However, this is especially challenging under exercise as usually multiple (at least 3) measurements have to be performed, and a stable hemodynamic condition is needed for a time of about 5 min.

Several studies have demonstrated the benefit of stress testing during RHC. It can be helpful in addition to the resting measurement to unmask latent left heart disease, to better understand the pathophysiology of the patients’ clinical symptoms such as dyspnea, and it even carries prognostic information (2–6). Dynamic exercise testing using a cycle ergometer has become clinically established for this purpose (7).

As this form of stress during RHC is not widely available, it has already been compared to volume loading with rapid infusion of 500 mL isotonic fluid regarding the effect on hemodynamic parameters. There was no agreement on the physiological effects on the cardiovascular system, but both types of loading could demonstrate to unmask latent diastolic dysfunction in different patient populations (8).

Another physical stress testing method, which could be used in a space-saving as well as cost-effective manner, is handgrip. This isometric type of contraction requires the patient to induce a load by constant fist closure, inducing an increase in left ventricular afterload and myocardial oxygen demand (9). It has been evaluated for its utility as cardiac stress test in different situations. In a study by Samuel et al., handgrip exercise was found to be an effective exercise method to assess the left ventricular diastolic function during echocardiography (9). Handgrip stress was also used in patients with functional mitral regurgitation during echocardiography, with some of the patients showing stress-induced changes that predicted survival and thus had a potential impact on the therapeutic approach (10). It was also used in patients with mitral regurgitation during RHC to assess the effect on PAWP increase (11).

The aim of the present study was to compare hemodynamic changes during exercise by cycle ergometry and isometric handgrip regarding equivalence and suitability to reveal latent left heart disease in patients with known or suspected PH.

## 2 Methods

### 2.1 Patients and study design

In this prospective single-center study, we planned to include 50 consecutive adult patients with clinical indication for RHC after giving written informed consent. As this was the first study investigating low-intensity handgrip exercise in this specific patient population, the hemodynamic effects of handgrip were difficult to predict, so no formal statistical power calculation could be performed. The study was approved by the local ethics committee (Project No 22-2811-101).

The examinations were performed between May and December 2022 in the RHC laboratory of the Department for Internal Medicine II, Pulmonology section, University Medical Center Regensburg (UKR), a national PH referral center. Hemodynamic measurements were carried out at rest and during both exercise modalities in a randomized order, separated by another resting phase. For randomization, the random function of Microsoft Excel, version 2208 (Microsoft Corporation, Redmond, Washington, United States), was used.

In case of patients’ inability to perform either type of exercise, patients were included in the analysis of the hemodynamic response compared to baseline but were excluded from the comparative analysis of the two exercise modalities.

PH was defined by a resting mPAP > 20 mmHg on RHC. According to the current guidelines and after thorough differential diagnosis, patients were assigned to groups 1–5 of the current PH classification (or ‘unclassified PH’ when pulmonary vascular resistance (PVR) was ≤ 2 Wood Units (WU) and PAWP ≤ 15 mmHg), respectively. An increase of PAWP from ≤ 15 mmHg at rest to > 25 mmHg was defined as exercise induced (latent) left heart disease (1).

### 2.2 Exercise modalities

For the handgrip exercise, the Martin Vigorimeter (Gebrüder Martin Company, Tuttlingen, Germany) was used. With this device, pressure is generated by squeezing a pear-shaped rubber ball connected to a manometer, which contains a pressure scale in kPa or bar. There are three different sizes available, which were individually selected for each patient depending on the size of the hand and grip comfort.

Before the examination, a maximum force test with the handgrip device was performed in each patient. For this purpose, the patients were instructed to compress the ball of the handgrip device three times with maximum force. The highest value was set as the maximum force value. As the maximum force can only be maintained for a very short time, which does not allow the collection of all hemodynamic parameters, and for standardization purposes, we chose 20% of the individual maximum force for the isometric exercise during the RHC. This intensity was selected based on tests in healthy volunteers (not reported) to ensure that patients were able to maintain the load continuously during the measurements including determination of CO by the thermodilution method. The maximal force test was performed before catheter insertion solely for calibration purposes and not as a hemodynamic measurement stage.

The cycle ergometry work rate was set to a constant value between 10and 50 watts based on the estimated exercise capacity according to patient’s history. We instructed the patients to maintain approximately 60 revolutions per minute throughout the examination.

### 2.3 RHC procedure

We performed RHC using a balloon-tipped catheter (Swan-Ganz, Edwards Lifesciences, Irvine, CA, United States), inserted either via the antecubital or the internal jugular vein, under fluoroscopic guidance if necessary. We positioned the patients in a semi-recumbent position with the upper body elevated to 45 degrees and adjusted the cycle ergometer to their height. The pressure transducer was leveled at the mid-chest level and zeroed to atmospheric pressure. All hemodynamic pressures, both at rest and during exercise, were measured at end-expiration. Cardiac output (CO) was determined by thermodilution, averaging at least three measurements with less than 10% deviation. PVR was calculated as (mPAP - PAWP)/CO. We monitored the patients’ electrocardiogram, pulse oximetry, and non-invasive blood pressure throughout the procedure.

After catheter insertion and at least 5 min of rest, the following parameters were measured at rest: systolic (sPAP) and diastolic pulmonary artery pressure (dPAP), mPAP (digital mean), PAWP, right atrial pressure (RAP), CO, non-invasive systolic (SBP), diastolic (DBP) and mean arterial pressure (MAP), heart rate (HR), mixed venous (S_*v*_O_2_) and peripheral oxygen saturation (S_*p*_O_2_) by blood gas analysis and pulse oxymetry, respectively.

After data collection at rest, the first of the two exercise procedures was started according to prior randomization. The measurements were started after at least 90 s of exercise when pressure values had reached a steady state. The same sequence was used at rest and under exercise (1. pulmonary artery pressures and RAP simultaneously, 2. PAWP, 3. CO, 4. HR, 5. S_*v*_O_2_/S_*p*_O_2_, 6. non-invasive blood pressure). After completion of the first exercise measurement, a resting interval was introduced to ensure the return of pulmonary pressures to the initial resting level which was documented accordingly. Then the second exercise method was applied with parameter collection as described.

After registration of all parameters, vasoreactivity testing or step oximetry was performed if indicated according to our standard procedures (not reported). With the final removal of the catheter, the examination was completed.

### 2.4 Statistical analysis

Statistical analysis of the data was performed using SPSS software (version 28.0, SPSS, IBM, Armonk, NY, United States). Paired-sample *t*-tests were performed to test for significant differences between the values obtained during rest and exercise as well as between the different exercise tests. To compare hemodynamic parameters between pre-capillary and post-capillary PH, the distribution of variables was assessed for normality using the Shapiro-Wilk test. The independent-samples *t*-test was used for normally distributed variables, while the non-parametric Mann-Whitney U test was performed for variables not normally distributed in at least one of the groups.

Furthermore, linear regression models to determine correlation as well as Bland-Altman diagrams to determine the agreement of mPAP and PAWP values between the two different exercise-types were used.

For all comparisons, a *p*-value of < 0.05 was considered statistically significant. No correction for multiple comparisons was applied.

## 3 Results

### 3.1 Patient characteristics

Between May and December 2022, 50 consecutive patients with established or suspected PH were included. Out of those, 27 were female (54%), and their mean age was 68 ± 12 years. The majority of patients (*n* = 47, 94%) had PH at rest. The PH phenotype was classified for each patient using these resting parameters. Accordingly, 9 patients (19.1%) were assigned to group 1, 14 (29.8%) to group 2, 10 (21.3%) to group 3, 7 (14.9%) to group 4, and 2 (4.3%) to group 5 according to the current PH classification system (1). Five patients (10.6%) were diagnosed with unclassified PH. Further anthropometric data and cardiovascular risk factors can be found in Table 1.

**TABLE 1:** patient characteristics.

Parameter	All (*n* = 50)
Female sex, n (%)	27 (54)
Age, years	68 ± 12 (38–87)
Height, cm	168 ± 9 (150–191)
Body weight, kg	78 ± 21 (42–128)
Body mass index, kg/m^2^	27,3 ± 6,7 (16,5–44,1)
Current/Previous smoker, n (%)	33 (66)
Arterial hypertension, n (%)	41 (82)
Hypercholesterolemia, n (%)	24 (48)
Diabetes mellitus, n (%)	13 (26)
Interstitial lung disease, n (%)	4 (8)
Chronic obstructive pulmonary disease (%)	13 (26)
History of deep vein thrombosis, n (%)	2 (4)
History of pulmonary embolism, n (%)	11 (22)
Atrial fibrillation, n (%)	16 (32)
Coronary heart disease, n (%)	18 (36)
Anticoagulation, n (%)	27 (54)

All metric data given as mean ± standard deviation (min-max). Categorical variables were expressed as frequency (percentage).

### 3.2 Exercise testing

In 47 patients, both types of exercise could be performed. The mean duration of exercise was 7 ± 2 min for handgrip and 6 ± 1 min for cycle ergometry. This duration was necessary to complete the full hemodynamic measurement protocol, including the measurement of stable CO via thermodilution. For the handgrip exercise, the average load was 9 kPa, and for the cycle ergometer load 26 watts with a frequency of approximately 60 revolutions per minute. Three patients had to be excluded from the comparison between the exercise modalities: in 2 patients, exercise (one method each) had to be terminated prematurely due to exhaustion, and one patient was unable to cycle due to a leg length difference. No complications occurred during the measurements.

Both handgrip and cycle ergometer stress induced significant consistent changes compared to rest in the majority of parameters with a lower magnitude during handgrip exercise (Table 2). During cycle ergometry, all parameters changed significantly compared to resting values. The mPAP and PAWP increased from 35 ± 12 mmHg to 49 ± 15 mmHg (*p* < 0.001) and from 13 ± 7 mmHg to 18 ± 6 mmHg (*p* < 0.001), respectively. CO, oxygen saturation, and other hemodynamic values also showed significant changes as given in Table 2. Under handgrip exercise, most parameters changed significantly as well while the magnitude was considerably smaller. The mPAP and PAWP increased to 39 ± 13 mmHg (*p* < 0.001) and 15 ± 7 mmHg (*p* < 0.001), respectively. Three parameters did not show statistically significant changes compared to rest: MAP (100 ± 15 mmHg at rest vs. 100 ± 17 mmHg, *p* = 0.546), PVR (5.0 ± 3.2 WU vs. 5.3 ± 3.5 WU, *p* = 0.102), and S_*p*_O_2_ (95 ± 4% vs. 94 ± 3%, *p* = 0.308).

**TABLE 2:** Hemodynamic parameters during rest and exercise.

Parameter	Rest	Cycle	Handgrip	*p*-value (cycle vs. rest)	*p*-value (handgrip vs. rest)	*p*-value (cycle vs. handgrip)
HR, 1/min	72 ± 14 (46–117)	91 ± 18 (58–130)	76 ± 14 (53–125)	< 0.001	<0.001	< 0.001
MAP, mmHg	100 ± 17 (63–139)	105 ± 18 (77–142)	100 ± 15 (76–143)	0.017	0.546	0.001
mPAP, mmHg	35 ± 12 (14–69)	49 ± 15 (24–85)	39 ± 13 (14–70)	< 0.001	<0.001	< 0.001
PAWP, mmHg	13 ± 7 (4–34)	18 ± 6 (9–33)	15 ± 7 (4–33)	< 0.001	<0.001	< 0.001
RA, mmHg	6 ± 4 (1–16)	13 ± 6 (–27)	8 ± 4 (1–18)	< 0.001	<0.001	< 0.001
CO, l/min	4.9 ± 1.5 (2.5–9.9)	6.4 ± 2.1 (2.6–12.3)	5.1 ± 1.8 (2.5–10.3)	< 0.001	0.049	< 0.001
PVR, WU	5.0 ± 3.2 (0.6–12.6)	5.5 ± 3.5 (0.7–14.2)	5.3 ± 3.5 (0.2–14.4)	0.029	0.102	0.165
S_*v*_O_2_,%	66 ± 7 (51–82)	38 ± 13 (18–66)	63 ± 8 (46–82)	< 0.001	<0.001	< 0.001
S_*p*_O_2_,%	95 ± 4 (88–100)	91 ± 6 (77–99)	94 ± 3 (87–100)	< 0.001	0.308	< 0.001
mED, min	/	6 ± 1 (4–11)	7 ± 2 (4–11)	/	/	/
mWR_*Cycle*_, Watt	/	26 ± 9 (10–50)	/	/	/	/
mP_*Handgrip*_, kPa	/	/	9 ± 2 (4–15)	/	/	/

All metric data given with mean ± standard deviation (min-max). HR, heart rate; MAP, mean arterial pressure; mPAP, mean pulmonary arterial pressure; PAWP, pulmonary arterial wedge pressure; RA, right atrial pressure; CO, cardiac output; PVR, pulmonary vascular resistance; WU, Wood Units; S_*v*_O_2_, mixed venous oxygen saturation; S_*p*_O_2_, peripheral oxygen saturation; Med, mean exercise duration; mWR, mean work rate; mP, mean (handgrip) pressure.

Comparing parameters between cycle ergometry and handgrip, significant differences were found in almost all measured values. The peak mPAP was 49 ± 15 mmHg during cycle ergometry compared to only 39 ± 13 mmHg during handgrip exercise (*p* < 0.001). Similarly, peak PAWP reached 18 ± 6 mmHg during cycling and only 15 ± 7 mmHg during handgrip (*p* < 0.001), and peak CO was 6.4 ± 2.1 l/min during cycling and 5.1 ± 1.8 l/min during handgrip (*p* < 0.001). Oxygen saturations also differed significantly between the types of exercise, with S_*v*_O_2_ decreasing to 38 ± 13% during cycling compared to 63 ± 8% during handgrip (*p* < 0.001), and S_*p*_O_2_ dropping to 91 ± 6% during cycling and to 94 ± 3% during handgrip (*p* < 0.001). Changes in PVR showed a similar pattern with an increase to 5.5 ± 3.5 WU during cycle ergometry and to 5.3 ± 3.5 WU during handgrip exercise (*p* = 0.165), even if the difference was not statistically significant.

### 3.3 Correlation and agreements of pressures

**FIGURE 1:**
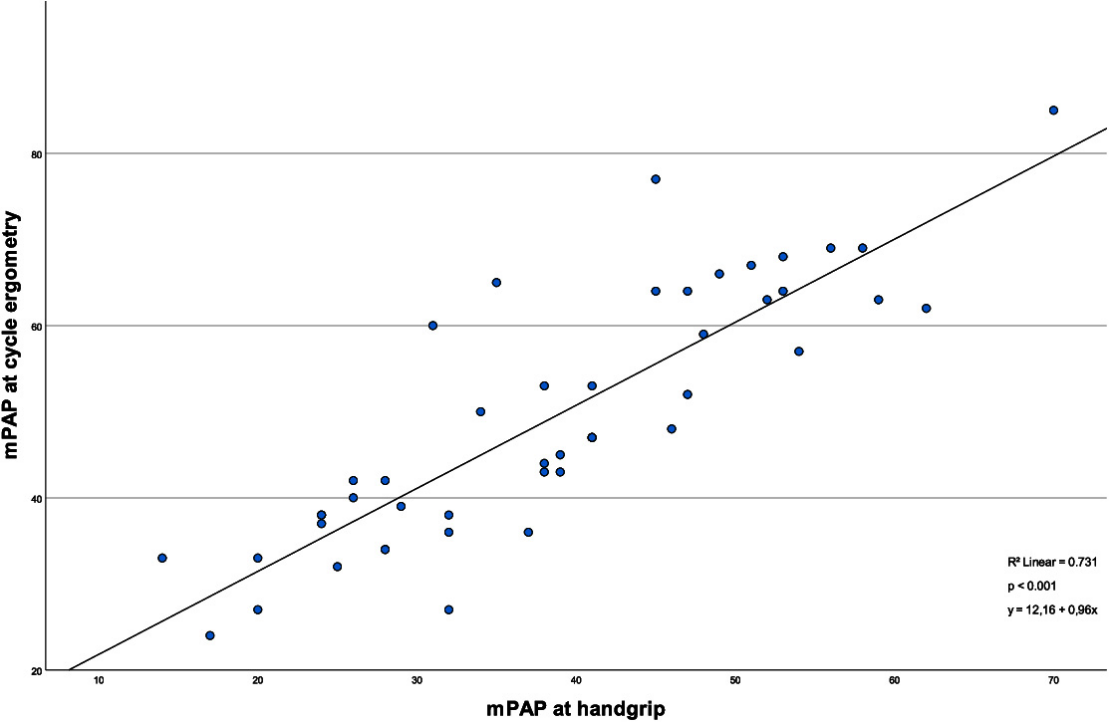
Correlation of mean pulmonary artery pressures between the types of exercise. This linear regression model illustrates the correlation of the mPAP values measured under the different exercise methods. The correlation between the different mPAP values is considered significant (*p* < 0.001).

**FIGURE 2:**
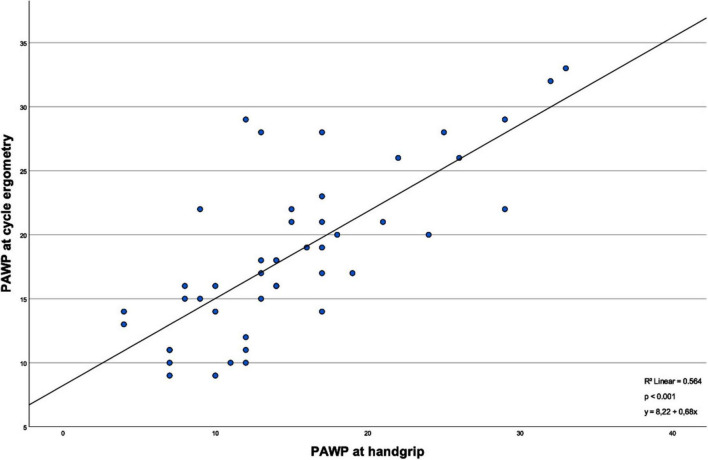
Correlation of pulmonary arterial wedge pressure values between types of exercise. This linear regression model illustrates the correlation of the PAWP values measured under the different exercise methods. The correlation between the different PAWP values is considered significant (*p* < 0.001).

As shown in Figure 1, the mPAP values between handgrip and cycle ergometry show a strong and significant positive correlation (*R*^2^ = 0.73, *p* < 0.001). The same is true for PAWP values during the different exercise types, illustrated in Figure 2 (*R*^2^ = 0.56, *p* < 0.001).

**FIGURE 3:**
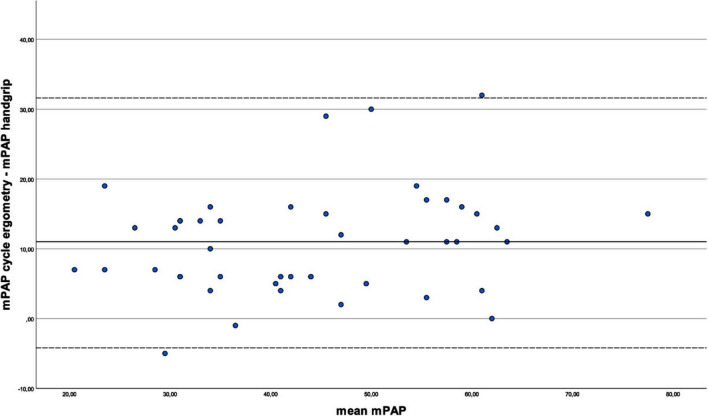
Comparison of mean pulmonary artery pressures between exercise types. In this Bland-Altman plot, the difference of the mPAP values during cycle ergometry and handgrip is plotted against the calculated mPAP mean values of the value pairs [(mPAP_*cycle*_ + mPAP_*handgrip*_)/2]. As the differences were not normally distributed, the median bias and percentile-based limits of agreement are presented. The median difference is 11.0 mmHg (mPAP under cycle ergometry - mPAP under handgrip), indicating a systematic bias of a higher mPAP under cycle ergometry compared to mPAP under handgrip. The 95% limits of agreement, corresponding to the 2.5th and 97.5th percentiles, are -4.2 mmHg and 31.6 mmHg. The plot shows a significant scatter around said median, with two values outside the limits of agreement.

**FIGURE 4:**
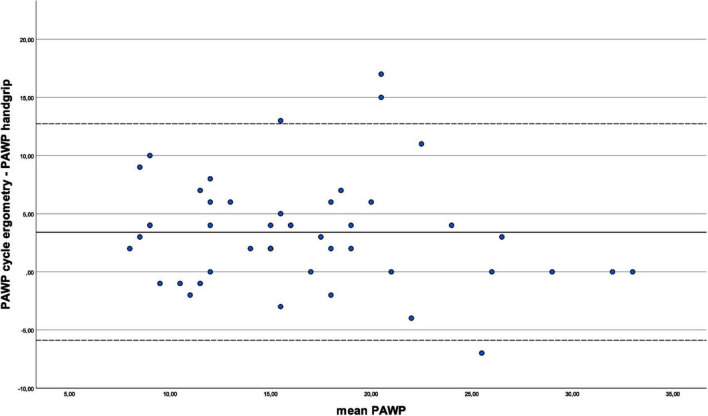
Comparison of pulmonary arterial wedge pressure (PAWP) between exercise types. In this Bland-Altman plot, the difference of the PAWP values during cycle ergometry and handgrip is plotted against the calculated PAWP mean values of the value pairs [(PAWP_*cycle*_ + PAWP_*handgrip*_)/2]. At a confidence interval of 95% the limits of agreement are –5.9 mmHg and 12.7 mmHg respectively (3.4 mmHg ± 1.96 × 4.8 mmHg). The mean difference is 3.4 mmHg (PAWP under cycle ergometry - PAWP under handgrip), indicating a systematic bias of PAWP under cycle ergometry over PAWP under handgrip. The plot also demonstrates a considerable variation around the mean difference, with four outliers exceeding the limits of agreement.

Figure 3 illustrates the agreement between the mPAP under cycle ergometry and handgrip stress tests. As the differences were not normally distributed, this analysis was based on the median. The median bias was 11.0 mmHg, indicating that cycle ergometry stress leads to systemically higher mPAP values than the handgrip stress. The plot does not show any systematic pattern or trend in the differences, suggesting that there is no relationship between the difference and the magnitude of pressures. Similarly, the PAWP measured under cycle ergometry was on average 3.4 mmHg higher than under handgrip as demonstrated in Figure 4. Again, there seems to be no correlation between difference and the magnitude of measurement.

### 3.4 Hemodynamic responses to exercise in pre- vs. post-capillary PH

For subgroup analysis, the patient cohort was stratified into pre-capillary (*n* = 29) and post-capillary (*n* = 13) PH based on resting hemodynamics. The post-capillary group consisted of patients with both isolated post-capillary and combined pre- and post-capillary PH. The hemodynamic profiles of these two groups were compared at rest and during both exercise modalities (Table 3).

**TABLE 3:** Hemodynamic comparison of pre-capillary vs. post-capillary pulmonary hypertension.

Parameter	PH group	Rest	Cycle	Handgrip
HR, 1/min	Pre-capillary	73 ± 15 (46–117)	90 ± 17 (58–124)	78 ± 15 (53–125)
	Post-capillary	68 ± 10 (54–90)	90 ± 22 (60–130)	70 ± 11 (55–91)
	*p*-value	0.349	0.943	0.090
MAP, mmHg	Pre-capillary	99 ± 15 (63–131)	105 ± 18 (77–137)	99 ± 15 (76–131)
	Post-capillary	102 ± 21 (74–139)	102 ± 17 (81–141)	100 ± 13 (88–127)
	*p*-value	0.620	0.602	0.781
mPAP, mmHg	Pre-capillary	38 ± 11 (22–69)	53 ± 13 (27–85)	42 ± 12 (20–70)
	Post-capillary	37 ± 9 (24–54)	51 ± 16 (27–77)	42 ± 10 (28–62)
	*p*-value	0.863	0.671	0.856
PAWP, mmHg	Pre-capillary	10 ± 3 (4–15)	16 ± 4 (9–29)	12 ± 4 (4–19)
	Post-capillary	23 ± 5 (16–34)	25 ± 6 (14–33)	24 ± 6 (15–33)
	*p*-value	< 0.001	<0.001	< 0.001
RA, mmHg	Pre-capillary	6 ± 4 (1–15)	13 ± 6 (6–27)	7 ± 4 (2–16)
	Post-capillary	9 ± 3 (3–16)	17 ± 4 (13–27)	11 ± 4 (6–18)
	*p*-value	0.012	0.033	0.002
CO, l/min	Pre-capillary	4.4 ± 1.0 (2.5–6.1)	5.7 ± 1.4 (2.6–7.9)	4.6 ± 1.2 (2.5–6.4)
	Post-capillary	5.3 ± 1.8 (2.8–8.2)	6.7 ± 2.6 (3.2–11.2)	5.6 ± 2.6 (2.7–10.3)
	*p*-value	0.111	0.213	0.236
PVR, WU	Pre-capillary	6.6 ± 3.0 (2.2–12.6)	7.0 ± 3.2 (1.7–14.2)	6.8 ± 3.4 (1.8–14.4)
	Post-capillary	3.1 ± 2.3 (0.6–8.1)	4.6 ± 3.6 (0.7–11.0)	3.9 ± 3.1 (0.2–11.2)
	*p*-value	< 0.001	0.046	0.006
S_*v*_O_2_,%	Pre-capillary	65 ± 7 (51–81)	35 ± 12 (18–63)	62 ± 8 (46–77)
	Post-capillary	64 ± 6 (53–75)	35 ± 12 (19–56)	60 ± 7 (47–71)
	*p*-value	0.658	0.970	0.547
S_*p*_O_2_,%	Pre-capillary	94 ± 3 (89–100)	89 ± 6 (77–99)	93 ± 3 (87–99)
	Post-capillary	96 ± 4 (88–100)	92 ± 5 (85–99)	97 ± 2 (93–100)
	*p*-value	0.374	0.189	0.007

All metric data given with mean ± standard deviation (min-max). HR, heart rate; MAP, mean arterial pressure; mPAP, mean pulmonary arterial pressure; PAWP, pulmonary arterial wedge pressure; RA, right atrial pressure; CO, cardiac output; PVR, pulmonary vascular resistance; WU, Wood Units; S_*v*_O_2_, mixed venous oxygen saturation; S_*p*_O_2_, peripheral oxygen saturation.

At baseline, patients with post-capillary PH exhibited significantly higher PAWP (23 ± 5 mmHg vs. 10 ± 3 mmHg; *p* < 0.001) and RA (9 ± 3 mmHg vs. 6 ± 4 mmHg; *p* = 0.012) compared to the pre-capillary group. Consequently, PVR was significantly lower in the post-capillary group (3.1 ± 2.3 WU vs. 6.6 ± 3.0 WU; *p* < 0.001).

During cycle ergometry, the significant differences between the groups persisted. PAWP (25 ± 6 mmHg vs. 16 ± 4 mmHg; *p* < 0.001), RA pressure (17 ± 4 mmHg vs. 13 ± 6 mmHg; *p* = 0.033), and PVR (4.6 ± 3.6 WU vs. 7.0 ± 3.2 WU; *p* = 0.046) all remained significantly different between the post-capillary and pre-capillary cohorts, respectively.

Similarly, during handgrip exercise, PAWP (24 ± 6 mmHg vs. 12 ± 4 mmHg; *p* < 0.001), RA (11 ± 4 mmHg vs. 7 ± 4 mmHg; *p* = 0.002), and PVR (3.9 ± 3.1 WU vs. 6.8 ± 3.4 WU; *p* = 0.006) maintained their significant differences. Additionally, during handgrip, S_*p*_O_2_ was found to be significantly higher in the post-capillary group (97 ± 2% vs. 93 ± 3%; *p* = 0.007). Other resting hemodynamic parameters did not differ significantly between the two groups.

### 3.5 Latent left heart disease

**FIGURE 5:**
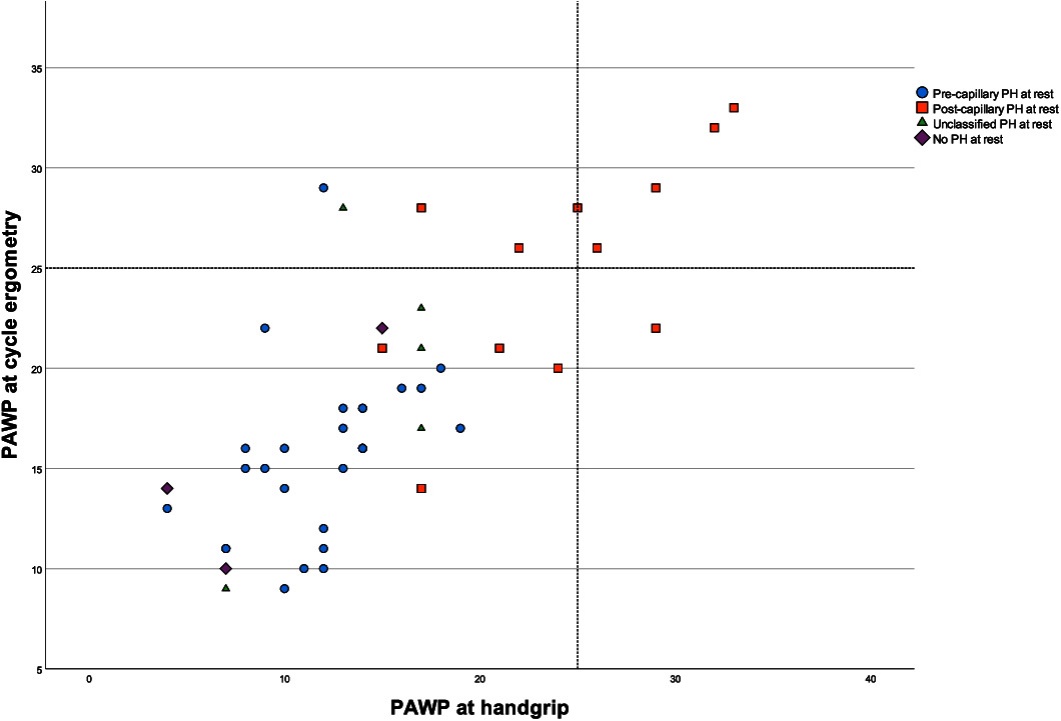
Comparison of pulmonary arterial wedge pressure (PAWP) values between cycle ergometry and handgrip. The cut-off for postcapillary pulmonary hypertension (PH) was set at 25 mmHg. Two patients with a PAWP ≤ 15 mmHg at rest showed an increase to > 25 mmHg during cycle ergometry exercise but not during handgrip exercise. The rest of the patients who had PAWP > 25 mmHG during either exercise modality already had a PAWP > 15 mmHg at rest, and belonged to the group of patients with postcapillary PH.

According to the current PH guideline, an increase in PAWP under exercise > 25 mmHg indicates left heart dysfunction (1). Figure 5 shows that only 2 patients with a resting PAWP ≤ 15 mmHg exceeded the cut-off of 25 mmHg during cycle ergometer exercise meaning a change in PH classification at rest, but not during handgrip exercise. In the remaining patients with precapillary PH, no left ventricular diastolic dysfunction was detected with any exercise modality. The rest of the patients who had a PAWP above 25 mmHg during exercise already had a resting PAWP above 15 mmHg and were classified as post-capillary PH already at rest, accordingly.

## 4 Discussion

### 4.1 Hemodynamic responses during exercise testing

In 50 patients with known or suspected PH undergoing RHC at rest and during exercise using cycle ergometer and low-intensity isometric handgrip, we found a significant increase in pulmonary arterial and systemic blood pressures, PAWP, and CO on exercise. Changes were significantly more pronounced under cycle exercise, which revealed an increase in PAWP > 25 mmHg in 2 patients with PAWP ≤ 15 mmHg at rest.

The alterations observed in hemodynamic parameters under stress were anticipated and align with the findings presented in other studies (12, 13). Müller et al. investigated the hemodynamic effects of cycle ergometry in 121 patients with pulmonary arterial hypertension (PAH) or chronic thromboembolic PH (CTEPH) and showed similar significant changes but different magnitudes (14). Notably, the mPAP increased on average by 24.2 mmHg in their study compared to only 14 mmHg in our patient cohort despite comparable baseline pulmonary pressures (mPAP 36.8 ± 11.8 mmHg versus 35.0 ± 12.0 mmHg in our study). On the other hand, the average PAWP increase was greater in our patients (5 mmHg vs. 2.9 mmHg). The different hemodynamic responses can be attributed to differences in methodology. While Müller and colleagues used an incremental stepwise cycle exercise protocol starting at 10 watts with increases up to 60 watts and beyond to individual maximum workload, we applied a constant workload of 26 ± 9 only. Furthermore, their patient population consisted of patients with PAH and CTEPH only, while our mixed patient population included almost one third group 2 PH patients. Finally, our patient population was slightly older (68 vs. 62 years).

Regarding handgrip exercise, we observed a considerably smaller PAWP increase in our patient population compared to other studies. In a study by Hamatani et al., a change in PAWP (ΔPAWP) of 11 mmHg was observed under handgrip stress, compared to only 2 mmHg in our patient cohort (11). This can be explained by their use of maximum force (100%) handgrip exercise for 2–3 min, compared to our submaximal, low-intensity (20%) approach. Moreover, only patients with moderate and severe mitral regurgitation were examined, who can be expected to have a higher left heart strain and thus a higher increase in PAWP even on low-intensity exercise. The differences in mPAP increase (10 mmHg vs. 4 mmHg in our study) can therefore be attributed to the different increase in PAWP. However, Hamatani and colleagues did not report CO measurements, limiting direct comparison of the overall hemodynamic response. In addition, it is hard to imagine that elderly patients with mitral regurgitation will be able to keep 100% handgrip force for 2–3 min. Further, a thorough CO determination is not possible in this short duration.

In this regard, the findings of Rommel et al. offer a valuable comparison. Their study in HFpEF patients using invasive pressure-volume loops during submaximal handgrip for 1 min showed a modest rise in cardiac index (3.0–3.4 l/min/m^2^) with significant increases in ventricular pressures (15). In contrast, our low-intensity protocol resulted in a minimal CO increase (ΔCO + 0.2 l/min) and no significant change in MAP (*p* = 0.546). Whereas the afterload challenge in the Rommel study was sufficient to unmask impaired ventricular relaxation and stiffness characteristic of HFpEF, our lower-intensity protocol was likely inadequate to provoke a comparable cardiovascular response. This highlights how the hemodynamic reaction to isometric exercise strongly depends on exercise intensity and patient phenotype. However, the method of CO measurement in their study was very complex and cannot be transferred to routine clinical practice.

### 4.2 Comparability of exercise modalities

In this study, designed to compare the effects of exercise by cycle ergometry and static low-intensity handgrip on hemodynamic parameters, we found statistically significant differences in all parameters except for PVR. While strong correlations between the exercise modalities were found for both mPAP (*R*^2^ = 0.73, *p* < 0.001) and PAWP (*R*^2^ = 0.56, *p* < 0.001), Bland-Altman analysis demonstrated systematic differences with consistently higher values during cycle ergometry. For the mPAP comparison, the median bias was 11.0 mmHg and for the PAWP comparison, the mean bias was 3.4 mmHg. For both parameters, the analysis showed wide limits of agreement but no systematic trends across the range of measurements. These findings indicate that although both exercise types induce changes of pulmonary pressures in the same direction, cycle ergometry provides a substantially greater hemodynamic challenge. This observation is supported by similar findings in other studies.

Wernhart et al. investigated different types of stress testing in patients at risk for heart failure with preserved ejection fraction, comparing both cycle ergometry and handgrip exercise similar to our approach. (16). Like in our study, they found significantly greater hemodynamic responses during cycle ergometry compared to handgrip exercise, with ΔPAWP, ΔmPAP, and ΔCO (also measured by thermodilution) increasing by 22.3 mmHg, 27.3 mmHg, and 3.3 l/min during cycling vs. 9.6 mmHg, 11.5 mmHg, and 1.9 l/min during handgrip, respectively. In comparison, our study showed more modest changes during cycle ergometry (ΔPAWP 5 mmHg, ΔmPAP 14 mmHg, ΔCO 1.5 l/min) and particularly during handgrip (ΔPAWP 2 mmHg, ΔmPAP 4 mmHg, ΔCO 0.2 l/min). The substantially higher hemodynamic responses in their study can be explained by distinct characteristics of their investigation: Their study population consisted of patients at risk for heart failure with preserved ejection fraction, which explains particularly the pronounced PAWP elevation during exercise. Additionally, they employed more intensive exercise protocols in both modalities—an incremental WHO-25 protocol for cycle ergometry with stepwise increases of 25W every 2 min until maximal subjective exertion (compared to our constant workload of 26 ± 9W), and dynamic handgrip contractions at 80% of maximum force for 1 min (compared to our isometric approach at 20% of maximum force). Although this study showed a decrease rather than an increase in PVR, both types of stress induced a similar change in PVR, without a significant difference between the values under the different exercise modalities (ΔPVR = 0.1 WU for both types of stress).

The different hemodynamic responses observed in our study are best explained by the fundamental physiological differences between the two exercise modalities. Cycle ergometry, a dynamic exercise involving large muscle groups of the lower limbs, primarily imposes a volume load on the heart by driving a substantial increase in CO to meet global metabolic demand (17). In contrast, isometric handgrip is a static exercise that activates a much smaller muscle mass in the forearm and primarily induces a pressure load by increasing systemic afterload, a process generally not accompanied by a notable rise in CO. Previous studies have demonstrated that such exercises can significantly elevate systemic arterial pressure (18, 19). However, a key finding of our study was the complete absence of an increase in blood pressure during our low-intensity handgrip protocol, suggesting that the stimulus was not sufficient to provoke the expected cardiovascular response. The greater stress caused by cycle ergometry compared to isometric handgrip exercise is underlined by the higher oxygen consumption, which is reflected by the significantly higher drop in S_*v*_O_2_.

PVR was the only parameter that did not differ significantly between the two exercise modalities ΔPVR = 0.5 WU during cycle ergometry and 0.3 WU during handgrip, *p* = 0.165). While PVR generally decreases under stress depending on age and health status (12, 17), our patient group showed a slight significant increase in PVR under cycle ergometry (ΔPVR = 0.5 WU). However, this result is consistent with other studies that also examined patients with PH (14, 20). PVR under stress can decrease, stay unchanged or increase in patients with cardiac or pulmonary diseases, depending on the workload and positioning (21).

### 4.3 Hemodynamic subgroup analysis between pre- and post-capillary PH

We also analyzed the distinct hemodynamic responses of pre- and post-capillary PH to both exercise modalities. As PAWP and PVR form the basis for differentiating these groups, significant differences between them were present at rest and maintained throughout both cycle ergometry and low-intensity isometric handgrip exercise (1). Additionally, RA was consistently and significantly higher in the post-capillary group during all conditions, reflecting the predominant right ventricular strain (‘phenotype’) due to chronic backward transmission of elevated left-sided filling pressures. This finding is consistent with existing literature (22). Despite these differences, no statistically significant differences were observed in mPAP or CO between groups at rest or during either exercise modality suggesting that under both isometric and dynamic exercise, different primary pathologies can result in a similar overall limitation of cardiopulmonary function.

The only exercise-specific significant difference was found in SpO_2_, which was slightly but significantly higher in the post-capillary group during handgrip exercise (97 ± 2% vs. 93 ± 3%; *p* = 0.007). However, the small sample size of the post-capillary cohort (*n* = 13) limits the robustness of this finding and hinders definitive physiological interpretation. While this modest SpO_2_ divergence could suggest subtle differences in gas-exchange responses or ventilation/perfusion matching between phenotypes, drawing firm conclusions will require larger studies with greater power and direct arterial blood-gas measurements to clarify its true clinical relevance.

### 4.4 Efficacy to unmask left heart disease

Based on the concept of an increase in left ventricular afterload by the application of isometric handgrip exercise, an elevation of PAWP was anticipated especially in those patients with a (latent) left ventricular diastolic dysfunction. In our examined cohort, only two PH patients with PAWP ≤ 15 mmHg at rest exceeded the cut-off of 25 mmHg in the context of cycle ergometer stress but not under handgrip stress. This could be explained by the PH etiology in our patients which was either clear PH due to left heart disease already at rest or another clear pre-capillary pathology (e.g., PAH, PH due to lung disease or CTEPH). However, also in line with the literature, our results suggest that cycle ergometer exercise is more sensitive than handgrip exercise for detecting latent left ventricular diastolic dysfunction (16). To identify patients with latent left ventricular diastolic dysfunction, other methods should be considered. Fluid challenge and passive leg raise are methods that lead to an increase in PAWP in patients with impaired left ventricular function due to the increased preload. Ewert et al. compared fluid challenge with cycle exercise during RHC in patients with heart failure with preserved ejection fraction and found that both methods could unmask diastolic dysfunction albeit in different patient groups. However, exercise testing elicited a more pronounced hemodynamic response, highlighting its potential as a robust diagnostic tool (8). Both fluid challenge and passive leg raise were also examined by Wernhart et al. and were found to be inferior to cycle exercise in this regard (16). This contrasts with the results of van de Bovenkamp et al. who successfully employed passive leg raise as a diagnostic adjunct for the identification or exclusion of latent left ventricular diastolic dysfunction (23). Further studies are needed to confirm those results.

While handgrip exercise has demonstrated utility in echocardiographic assessments of left cardiac dysfunction within specific cohorts in previous studies (9, 10), it seems to be inferior to cycle exercise in RHC in this regard, at least when using a static stress of 20% of the maximum force.

### 4.5 Limitations

The main limitation of our study is the heterogeneity of PH etiologies. Therefore, our findings may not be generalizable to all PH patients. Future studies with larger and more homogeneous samples (e.g., enriched with risk factors for left heart disease) are needed to confirm our results.

This heterogeneity also limited the discriminatory power of flow-corrected mPAP/CO and PAWP/CO slopes, which were calculated but are not included in this manuscript, as they did not demonstrate robust differentiation between pre- and post-capillary PH in our small cohort.

Moreover, the intensity of 20% of the maximum force for the handgrip stress, also chosen for practical reasons and standardization, seemed to be too low. Due to the individual difficulty of the examination, the length of the measurements and the reaching of a steady state, the duration of the examination as well as the exercise duration slightly differed for each patient. In some cases, this led to brief exercise interruptions during the handgrip stress due to exhaustion, which could have affected the measured values.

CO was assessed by thermodilution, which is more accurate compared to indirect Fick measurements but takes significantly longer, being the main reason for the low intensity of handgrip stress in our study. However, we believe that CO measurements, crucial for a full hemodynamic assessment, by the direct Fick method or by the use of flow-volume-loops are unfeasible in daily clinical practice.

Pressure measurements were performed during the end-expiratory phase which may have led to an overestimation of true exercise-induced hemodynamic changes due to respiratory fluctuations.

In addition, in the absence of follow-up information no possible association with prognosis of our findings could be assessed.

## 5 Conclusion

Isometric handgrip exercise at a low intensity of 20% of maximal force leads to significantly smaller changes in hemodynamic parameters on RHC in patients with PH than cycle ergometry. Thus, it can’t be used as an adequate substitute for the established exercise method. Furthermore, our findings suggest that cycle ergometry is more sensitive than static low-intensity handgrip exercise in unmasking left heart disease in patients with no or precapillary PH at rest during RHC and should therefore be preferred as a diagnostic tool. For handgrip exercise to serve as a meaningful diagnostic tool for assessing cardiopulmonary hemodynamics, a protocol with a higher intensity appears to be required, however carrying the challenge of reliable and quick CO determination on exercise.

## Data Availability

The raw data supporting the conclusions of this article will be made available by the author, without undue reservation.
